# Microbial determinants of arthritis

**DOI:** 10.17179/excli2020-3180

**Published:** 2020-12-09

**Authors:** Shakti D. Shukla, Madhur D. Shastri, Niraj Kumar Jha, Kamal Dua

**Affiliations:** 1 Priority Research Centre for Healthy Lungs and School of Medicine and Public Health, Faculty of Health and Medicine, University of Newcastle, Callaghan, NSW, Australia; 2 School of Pharmacy and Pharmacology, University of Tasmania, Hobart, Australia; 3 Department of Biotechnology, School of Engineering & Technology (SET), Sharda University, Greater Noida, 202310, UP, India; 4 Discipline of Pharmacy, Graduate School of Health, University of Technology Sydney, Ultimo, NSW 2007, Australia

## ⁯⁯⁯⁯

***Dear Editor,***

Arthritis is an umbrella term encompassing over 100 medical conditions that affect the musculoskeletal system. Arthritis-related problems include pain, stiffness, inflammation and damage to joint cartilage and surrounding structures. This can result in joint weakness, instability and deformities leading to disability and chronic pain. In Australia, approximately 3.8 million Australians suffer from arthritis and the costs associated with the management of disease is more than $ 2.1 billion each year in medical care and indirect costs (Ackerman et al., 2018[2]). Arthritis is not yet curable, and although the disease could be managed pharmacologically, it appreciably affects the patient's quality of life and includes varying degrees of discomfort and pain. One form of the reactive arthritis is spondyloarthritis (SpA), which is characterized by arthritis, enthesitis, inflammation of the axial skeleton, conjunctivitis, and a pustular psoriasis-like skin rash called keratoderma blenorrhagica (Rich et al., 1996[12]). The mechanism of reactive arthritis is poorly understood, but anti-tumor necrosis factor (TNF) therapy has been shown to be a safe and effective treatment (Meyer et al., 2011[8]).

Several microbial infections are also shown to be associated with an onset of arthritis. Bacterial pathogens, such as *Staphylococcus aureus*, *Streptococcus spp.,* *Pseudomonas aeruginosa* and *Escherichia coli* are isolated from patients with infection-induced arthritis (Shirtliff and Mader, 2002[13]). Bacteria-induced or aggravated arthritis is generally referred to as septic arthritis and usually affects individuals aged over 60 years. Moreover, approximately 10 % of cases are polymicrobial in nature (Shirtliff and Mader, 2002[13]). Crucially, the lack of a defined synovial membrane allows an easy access to the invading bacteria leading to successful colonization and subsequent inflammation of the synovium. The major risk factors for bacterial entry includes pre-existing joint or skin condition, recent injury or surgery, bacteremia, compromised immune system and acute or prolonged corticosteroid regimen (Perry, 1999[10]). Once bacteria colonize the host joints, the host immune system is activated which is characterized by increased levels of pro-inflammatory cytokines, including interleukin (IL)-1β, IL-6, IL-8 and tumor necrosis factor-alpha (TNF)-α (Osiri et al., 1998[9]). Further, the activation of T-cells, especially CD4+ cells has been shown to play a key role in the pathogenesis of *S. aureus* induced arthritis (Abdelnour et al., 1994[1]). The most common treatment includes antibiotic administration and surgery to drain the purulent fluid from the affected joints. Albeit less prevalent (~1 %), the viral pathogens, such as parvovirus B19, hepatitis virus (B and C), HIV and the alphaviruses are significantly associated with viral-induced acute arthritis that could last from several days to months (Marks and Marks, 2016[7]). The duration and severity of virally induced arthritis seems to be associated with age (> 60 years) and viral load in blood (> 10^10^ virus/ml) on the patients (Hoarau et al., 2010[6]). Moreover, increased expression of IFN-α mRNA in peripheral blood mononuclear cells, and serum IL-12 was recorded in those who exhibited more chronic arthritis symptoms (12 months post infection) (Hoarau et al., 2010[6]).

The recent interest in overall microbial communities and functionalities, termed as microbiome, and its role in the progression and severity of chronic inflammatory diseases has gained considerable traction (Budden et al., 2019[5]; Alemao et al., 2020[3]; Vemuri et al., 2018[14]). Previous reports indicate that the host genetics influences microbial community within the host, as well as there exists a considerable relationship between host microbiota and organ inflammation in (SpA), a heritable condition affecting 2 % of the population and include ankylosing spondylitis (AS), psoriatic arthritis, reactive arthritis, uveitis, and arthritis associated with inflammatory bowel disease (IBD) (Rehaume et al., 2014[11]). This has been investigated in animal models of SpA. For instance, BALB/c ZAP-70W163C-mutant (SKG) mice (susceptible to SpA), Toll-like receptor 4 (TLR-4)-deficient SKG mice, and wild-type BALB/c mice were housed under specific pathogen-free conditions and some of these mice were recolonized with altered Schaedler flora. All of the mice were injected intraperitoneally with microbial β-1,3-glucan (curdlan). The authors found that microbiota content and response to curdlan varied according to whether T-cell receptor signal strength was normal or was impaired due to the *ZAP-70W163C* mutation. Moreover, curdlan triggered acute inflammation regardless of the presence of the SKG allele or microbiota. However, the severity of arthritis was not or minimally attenuated by microbiota. Notably, the host microbial composition was crucial in ileal IL-23 expression, ER stress, lymph node IL-17A production, goblet cell loss, and ileitis development. TLR-4 deficiency and microbiota transfer (co-housing SKG mice with wild-type BALB/c mice) were found to suppress ileitis but not arthritis (Rehaume et al., 2014[11]). In another animal study, the vaginal infection with live *Chlamydia muridarum* resulted in the development of arthritis, spondylitis, and psoriasiform dermatitis in female SKG mice, but not in BALB/c mice (Baillet et al., 2015[4]). Interestingly, the production of interferon-γ and interleukin-17 was impaired in T cells from SKG mice but tumor necrosis factor (TNF) responses were exaggerated, compared to findings in T-cells from BALB/c mice. Unlike previous observations in arthritis triggered by b-glucan (Rehaume et al., 2014[11]), no autoantibodies developed. 

In summary, microbial infections in general and microbiota more recently, have demonstrated an association with arthritis progression and severity. Further, focused studies are needed to underpin the mechanisms linking specific microbial species to the inflammatory processes in arthritis, both at the onset and progression stages to develop targeted, microbial-based therapies for treating and preventing arthritis.

## Conflict of interest

None.

## Authors’ contribution

Conceptualization: SDS, KD; Writing, reviewing and revisions: all authors; final approval: all authors.

## Funding

None to declare.
